# Current Genomics in Cardiovascular Medicine

**DOI:** 10.2174/138920212802510484

**Published:** 2012-09

**Authors:** Vinit Sawhney, Scott Brouilette, Dominic Abrams, Richard Schilling, Benjamin O’Brien

**Affiliations:** 1 Department of Cardiology, St Bartholomew’s Hospital, London, U.K; 2 William Harvey Research Institute, Queen Mary University London, U.K; 3 Partek Inc, St Louis, Missouri, USA; 4 Department of Cardiology, Children’s Hospital, Boston, USA; 5 Department of Intensive Care Medicine and Cardiac Anaesthesia, St Bartholomew’s Hospital, London, U.K; 6 Department of Anaesthesiology, Berlin Heart Institute, Berlin, Germany; 7 Life Sciences Division, E.O. Lawrence Berkeley National Laboratories, Berkeley, California, USA

**Keywords:** Cardiovascular disease, GWAS, Gene sequencing, Personalised medicine.

## Abstract

Cardiovascular disease (CVD) is a heterogeneous, complex trait that has a major impact on human morbidity and mortality. Common genetic variation may predispose to common forms of CVD in the community, and rare genetic conditions provide unique pathogenetic insights into these diseases. With the advent of the Human Genome Project and the genomic era, new tools and methodologies have revolutionised the field of genetic research in cardiovascular medicine. In this review, we describe the rationale for the current emphasis on large-scale genomic studies, elaborate on genome wide association studies and summarise the impact of genomics on clinical cardiovascular medicine and how this may eventually lead to new therapeutics and personalised medicine.

## INTRODUCTION

Cardiovascular disease: CVD is a heterogeneous, complex trait that has a major impact on human morbidity and mortality. The field of cardiovascular medicine involves a broad spectrum of abnormalities that are characterised by various clinical and etiological features – the major categories being coronary artery disease, congenital heart disease, heart muscle disorders and conduction disorders. Despite tremendous progress in knowledge gained, CVD remains the leading cause of death in the United Kingdom [1] and it has overtaken infectious diseases as the leading cause of death worldwide [2]. Increased life expectancy has generated an unprecedented growth in the older segment of the population, with an accompanying rising burden of aging-associated cardiovascular disorders. CVD is heritable and a two generational history of CVD remains a major risk factor, therefore genetic studies have long been pursued to elucidate the underlying disease mechanisms. However, CVD is not a homogenous phenotype and its etiologic complexity has been a formidable challenge. Common genetic variation may predispose to common forms of CVD in the community, and rare genetic conditions provide unique pathogenetic insights into these diseases. Public availability of vast amounts of detailed sequence information about the human genome, completed sequence data on other animal genomes, and private sector development of high-throughput genetic technologies has transformed in a few short years the conduct of cardiovascular genetics and genomics research from a primary focus on Mendelian disorders to a current emphasis on genome-wide association studies: GWAS.

However, the clinical practice of cardiovascular medicine is largely governed by a phenotype based approach. Nevertheless, functional genomics has led to an improvement of our understanding of CVD and can be translated to clinical utility. Gene-based pre-symptomatic prediction of illness, finer diagnostic sub-classifications and improved risk assessment tools will permit earlier and more targeted intervention. Pharmacogenetics will guide our therapeutic decisions and monitor response to therapy. Personalised medicine requires the integration of clinical information, stable and dynamic genomics and molecular phenotyping.

In this review, we describe the rationale for the current emphasis on large-scale genomic studies, elaborate on genome wide association studies and summarise the impact of genomics on clinical cardiovascular medicine and how this may eventually lead to new therapeutics and personalised medicine.

## GENOME WIDE ASSOCIATION STUDIES (GWAS)

We have accepted that CVD is a complex genetic trait showing familiality but no precise mode of inheritance. However, in the “pregenome era” pedigree studies have provided evidence for Mendelian mode of transmission for rare familial CVD, ion channel dysfunction underlying long QT syndrome, and sarcomeric and cytoskeletal protein abnormalities underlying hypertrophic and dilated cardiomyopathies. However, these mutations only account for a minority of the genetic basis of cardiovascular diseases [2-4]. It is now possible to systematically search the entire human genome for common variants that are associated with a particular phenotype. This is largely due to the advances in large scale genotyping after the completion of the Human Genome Project in 2003 [5, 6] and the Hap Map in 2005 [7, 8]. But leveraging this wealth of genetic information relies on the principle of Linkage Disequilibrium: LD is used to identify a set of common variants in the human genome that are excellent statistical proxies for genetic variation at a particular frequency [9].

GWAS have become more popular in recent years. Their rise can be attributed to the availability of whole genome Single Nucleotide Polymorphism (SNP – variant DNA sequence due to change in a single nucleotide) panels (e.g. the Illumina 1 M and Affymetrix 6.0 arrays, each of which can scan >1 million SNPs), large-scale genotyping technologies that are available at multiple genetics centres, the rapidly dropping sequencing cost per genome and powerful association analysis methods and accompanying software [10, 11].

GWAS are case-control studies, wherein research subjects are typed for a large number of SNPs, typically 3, 000,000 to 10,000,000, and the allele or genotype frequencies are evaluated for differences between groups or for correlations with continuous traits. GWAS are primarily designed to provide an unbiased survey of the effects of common genetic variants. The power of the GWAS to detect the phenotype associated alleles depends directly on the sample size of the study population, MAF (Minor allele frequency i.e. the frequency at which the less common allele of the SNP occurs in the population), strength of LD between the markers, the causal variants and the effect sizes of the alleles.

At present, 3 conditions must be satisfied to be considered susceptible loci through GWAS: (1) sufficient sample size in the genome-wide scan (at least 1000 each of cases and controls), (2) association *P*-value at the genome-wide significance level (*P*< 5 x 10^-8^) and (3) confirmation of the association by independent replication studies [12].

### GWAS and Coronary Artery Disease

It has been estimated that heritable factors account for 30%–60% of the inter-individual variation in the risk of coronary artery disease: CAD [13]. Genome-wide association studies have identified several common variants that associate with risk of CAD [14]. Recently, a meta– analysis of 14 GWAS of coronary artery disease comprising 22,233 individuals with CAD (cases) and 64,762 controls of European descent was completed, followed by genotyping of top association signals in 56,682 additional individuals. This analysis identified 13 loci newly associated with CAD at *P *< 5 × 10−8 and confirmed the association of 10 of 12 previously reported CAD loci. The 13 new loci showed risk allele frequencies ranging from 0.13 to 0.91 and were associated with a 6% to 17% increase in the risk of CAD per allele [15]. A recent genome wide association analysis of 2078 CAD cases and 2953 control subjects, identified 950 single nucleotide polymorphisms: SNPs associated with CAD at p < 10 ^-3^. Subsequent use of data on genetic variants and addition of data on global monocytic gene expression revealed a novel association signal at chromosome 10q 23.31 within the LIPA: lysosomal lipase acid A gene. An assessment of LIPA SNPs and transcript with cardiovascular phenotype revealed an association of LIPA transcript levels with impaired endothelial function [16]. The GWAS loci associated with CAD are shown in (Table **1**).

### GWAS and QT/QRS

The QT interval represents the time for both ventricular depolarisation and repolarisation to occur and hence estimates the duration of ventricular action potential. Long QT syndrome is due to prolonged repolarisation of the ventricular myocyte, manifested as a prolonged QT interval on the ECG that predisposes to ventricular tachycardia in the form of torsade des pointes, ventricular fibrillation and sudden cardiac death. Long QT syndrome is typically an isolated, autosomal dominant condition with significant variability in disease penetrance even in those harbouring the same disease-causing mutation, although the condition may be associated with sensorineural deafness and a more severe cardiac phenotype when inherited in a recessive fashion. However, the genetic mutations identified in the Mendelian form explain < 25% of the heritability. 

QT-interval length on ECG was among the first CVD phenotypes to be studied by GWAS, revealing a strong association with SNPs in the *NOS1AP *gene (1q23.3) in a staged study that involved 2 independent cohorts and was subsequently independently replicated in another large cohort [24, 25]. Unlike most genes known to be associated with QT-interval length, *NOS1AP *does not encode an ion channel but rather is a regulator of nitric oxide synthase; its mechanism of prolonging the QT interval is unknown. Moreover, 9 additional loci along-with NOS1AP have been associated with QT duration by the QTSCD and QTGEN consortium [26, 27], which has led to the identification of new candidate genes with ventricular arrhythmias and sudden cardiac death. 

The electrocardiographic QRS interval reflects ventricular depolarisation, and its duration is a function of electrophysiological properties within the His-Purkinje system and the ventricular myocardium. A diseased ventricular conduction system can lead to life-threatening brady-arrhythmias, such as heart block, and death in the general population and in cohorts with hypertension and coronary artery disease [28-30]. In a population-based study, prolonged baseline QRS was associated with incident heart failure. A significant association between QRS duration and risk of congestive cardiac failure was observed. Incomplete and complete bundle branch block were associated with a 1.5- and 2- fold risk of congestive cardiac failure respectively [31].

Twin and family studies suggest a genetic contribution to QRS duration, with heritability estimates of up to 40% [32, 33]. Candidate gene and genome-wide studies identified a limited number of loci associated with QRS duration, supporting the hypothesis of the contribution of common genetic variation in QRS duration [34-36]. 

A recent meta-analysis of 14 GWAS consisting of 40,407 individuals of European descent with additional genotyping in 7170 Europeans yielded genome wide significance associations of QRS duration with common variants in 22 loci. Variation in four of these loci (locus 1, *SCN5A-SCN10A*; locus 2, *CDKN1A; *locus 8, *TBX5; *and locus 21, *DKK1*) were previously associated with QRS duration in smaller independent studies using both candidate gene and genome-wide approaches [34-36]. The 22 loci include genes in a number of interconnected pathways, including some previously known to be involved in cardiac conduction, such as sodium channels, calcium-handling proteins and transcription factors, as well as previously unidentified processes not known to be involved in cardiac electrophysiology, such as kinase inhibitors, growth factor-related genes and others. They also demonstrated that *SCN10A*, a candidate gene at the most significantly associated locus in this study, is expressed in the mouse ventricular conduction system, and treatment with a selective *SCN10A *blocker prolongs QRS duration. GWAS loci associated with QRS duration are illustrated in (Table **2**).

It has also been shown in previous studies that in addition to their association with QRS duration, variants in *SCN5A *and *SCN10A *are associated with atrial conduction (PR interval) as well as atrial and ventricular fibrillation [35, 36, 38]. These results emphasise the crucial role played by these genes in cardiac conduction and the generation of arrhythmias. 

### GWAS and Stroke Susceptibility

Stroke is the leading cause of morbidity and mortality in the developed world. It can be broadly classified into ischaemic (cardio-embolic, large artery atherosclerosis or small vessel disease) and haemorrhagic stroke; with ischaemic stroke responsible for >80% of the cerebrovascular events.

In the first successful GWAS for ischaemic stroke [39] the only signal to meet genome-wide significance was a SNP near PITX2 (paired-like homeodomain transcription factor 2), which had previously been associated with atrial fibrillation: AF [40]. AF, which is characterised by chaotic electrical activity of the atria, is one of the most common forms of electrical instability and is thought to be responsible for the majority of CES (cardio-embolic stroke) events [41]. Intriguingly, while the association was strongest for CES, the SNP was also associated with non-cardiogenic stroke, suggesting that this is due to a misclassification of the cause of stroke as a result of undiagnosed AF. Given the higher risk of recurrence in patients with CES, and greater morbidity and mortality, this might have important clinical implications [42]. Two additional GWAS for stroke have been published, one focused on intracranial aneurysm [43], a major cause of haemorrhagic stroke, the other examined all strokes [42].

Bilguvar and colleagues [42] identified three loci associated with intracranial aneurysm, including the CDKN2A CDKN2B locus, which have previously been implicated in CAD (described above). Of the two novel loci identified, one (located on Ch 2q) is in a region containing four genes, and thus the relevant gene is unclear. The second locus (on Ch 8q) is particularly intriguing, as there seem to be two independent association signals flanking a single gene, SOX17 (SRY-box 17). The idea that associated genes are likely to harbour multiple independent variants is gaining support [45, 46] and suggests that novel analytical tools that account for this effect can increase power to identify genes through GWAS. The authors note that SOX17 is of particular interest because it is required for endothelial formation and maintenance, and Sox17-/- mice show vascular abnormalities [47]. In the second study, Ikram and colleagues identified a single locus, NINJ2, associated with stroke [42]. NINJ2 encodes ninjurin2, an adhesion molecule that is up- regulated in response to nerve injury. Analyses stratified by specific stroke subtype indicate that this locus is specific for ischaemic stroke, which accounts for more than 3/4^th^ of stroke cases.

### GWAS and CVD Risk Factors

Given the complex aetiology of CVD, the study of cardiovascular risk factors is likely to facilitate the elucidation of cardiovascular risk. Association studies have targeted multiple unrelated phenotypes measured in epidemiological surveys. Given that effect-sizes for variants identified through GWAS were smaller than previously suspected, many groups have now combined GWAS scans to identify novel loci for these quantitative traits [23, 40, 48-52].

In two co-published papers, analysis of lipid concentrations in a combined total of 8 816 individuals [50] with follow-up of >10 000 samples in each study, identified 19 loci associated with various lipid traits. More recently, two GWAS for lipid levels, each with >20 000 individuals, have lead to the identification of 35 total loci influencing lipid levels [51]. Kathiresan and colleagues reported a total of 30 loci influencing LDLC, HDL-C and/or TG. They detected multiple independent hits at some of the loci, resulting in 37 independent signals; the proportion of explained variance in each trait was 9.3% for HDL-C, 7.7% for LDL-C, and 7.4% for TG. In addition to LDL-C, HDL-C, and TG, Aulchenko and colleagues [51] looked for association with total cholesterol (TC). Overall, they identified 22 loci explaining up to 4.8% of the variance in each individual lipid trait (4.8% HDL-C, 3.4% LDL-C, 3.0% TG). Using prospective data, Aulchenko and colleagues evaluated the clinical relevance of genetic ‘risk profiles’, in particular, the TC risk profile, and demonstrated an improvement in CHD risk classification beyond traditional clinical factors of lipids, age, BMI, and sex. In a GWAS study of 100184 individuals of European ancestry (typed for 2.6 million SNP), 22 loci were associated with plasma LDL-C, 31 loci were associated with high-density lipoprotein cholesterol, and 16 loci were associated with triglycerides.These variants accounted for >25% to 30% of the genetic variance for each trait [53].

To date, GWAS have identified nearly 40 susceptibility loci for type 2 diabetes: T2D in European and Asian populations. The first GWAS for T2D was conducted in a French cohort composed of 661 cases and 614 controls, covering 392,935 SNP loci. This study identified novel association signals at *SLC30A8*, *HHEX*, *LOC387761*, conand *EXT2 *and validated the previously identified association at *TCF7L2* [54].

The first round of European GWAS confirmed 8 T2D susceptibility loci across multiple ethnic groups: *TCF7L2*, *SLC30A8*, *HHEX*, *CDKAL1*, *IGF2BP2*, *CDKN2A*/*B*, *PPARG*, and *KCNJ11*. In addition to these 8 loci, the WTCCC/UKT2D study identified a strong association between *FTO *variants and T2D, although the effect of *FTO *variants on conferring susceptibility to T2D was mostly mediated through increase in body weight [55]. Most of the T2D genetics cohorts have now combined to form DIAGRAM+, which yields an effective sample size of more than 22,000 subjects of European origin. In a recent study, 2,426,886 imputed and genotyped autosomal SNPs, with additional interrogation of the X-chromosome, were examined for association with T2D as a categorical phenotype. Twelve new loci were identified as susceptibility loci for T2D with a genome wide significance association (P < 5 x 10-8) [56].

A GWAS of systolic and diastolic blood pressure, which used a multi-stage design in 200,000 individuals of European descent identified 16 novel loci: six of these contain genes previously known or suspected to regulate blood pressure (GUCY1A3-GUCY1B3, NPR3-C5orf23, ADM, FURIN-FES, GOSR2, GNAS-EDN3) and the other ten provide new clues to blood pressure physiology [57].

### GWAS and Aortic Aneurysm

Thoracic aortic aneurysms and dissections: TAAD can be inherited as a single gene disorder, but the genetic predisposition in majority of the people is poorly understood. A recent multi-staged GWAS, compared 765 individuals with sporadic TAAD with 874 controls. The study identified common SNPs at 15q21.1 locus that associated with sporadic TAAD and attained genome wide significance. 107 SNPs associated with sporadic TAAD with p < 1x 10 ^-5 ^were then followed up in two separate sporadic TAAD cohorts.The associated SNPs were found in a large region in linkage disequilibrium encompassing FBN1, which encodes fibrillin. FBN1 mutations cause Marfan syndrome, whose major cardiovascular complication is sporadic TAAD. The study suggested that common genetic variants at 15q 21.1, (that probably act via FBN1) are associated with sporadic TAAD, suggesting a common pathogenesis of aortic disease in Marfan syndrome and sporadic TAAD [58].

### GWAS: Limitations

Although GWAS have been very useful in identifying a large number of phenotype associated alleles, that may lead to novel pathways involved in the pathogenesis of the phenotype, they do have some rather important limitations. 

The results of GWAS are subject to multiple-hypothesis testing. This is because a very large number of SNPs are typed to look for possible associations and hence this needs to be corrected for the possibility of random associations in statistical analysis. In addition, often the same study population is analysed for the association of the genotypes with multiple phenotypes, which also increases the likelihood of spurious associations. 

Despite the very large number of phenotype- associated alleles identified by GWAS, the number of functional SNP associated alleles is scarce [59]. Hence the results have not been useful in the immediate elucidation of the responsible mechanisms behind the observed genetic association. Therefore, GWAS need to be complemented with mechanistic studies to unravel the biological mechanisms responsible for genetic association, rather than simply increasing sample size to increase the power of the study.

Alleles identified in GWAS are often not the true causative alleles but are likely in linkage disequilibrium with the true causative alleles. The precise identification of gene and causal variants in complex diseases is difficult. The most challenging aspect is that most associated SNPs map to non-coding segments of the genome; although these regions contribute to complex diseases they contain genetic motifs that we do not fully recognise, and thus do not necessarily identify a specific gene [60]. Targeted re-sequencing of the region in linkage disequilibrium to locate the region of true genetic variation is required. Thus, extensive additional studies are typically required to complement the results of GWAS to identify the disease causing alleles.

The results of GWAS have minimal to modest impact, if any, on preclinical diagnosis, risk stratification, or genetic based prevention and treatment at an individual level. This could be due to large number of common variants with low magnitude of effect, rare variants with large effects, interaction between alleles at homologous loci and between alleles at non-homologous loci, epigenetic effects and underestimation of the effect of shared environment among relatives leading to inflated estimation of heritability [61]. Also, there is a need to integrate data at multiple levels i.e. genetic variations along-with epigenetic profiles. This approach will become more attainable with the advent of “3^rd^ Generation” sequencing technology (see below).

## GENE SEQUENCING

By their very nature, GWAS focus on a very small percentage of the total genome, thus there is great potential to miss the causative variations, irrespective of whether these are in coding or non-coding regions. However, capturing all possible variation within a sample requires a sequencing strategy. 

### Current Technologies

Since first introduced to the market in 2005, next-generation sequencing technologies have had a tremendous impact on genomic research. The next-generation technologies have been used for standard sequencing applications, such as genome sequencing and re-sequencing, and for novel applications previously unexplored by Sanger sequencing. 

The landmark publications of the late 1970 s by Sanger's and Gilbert's groups and notably the development of the chain termination method by Sanger and colleagues [62] established the groundwork for decades of sequence-driven research that followed. The chain-termination method published in 1977, also commonly referred to as Sanger or dideoxy sequencing, has remained the most commonly used DNA sequencing technique to date and was used to complete human genome sequencing initiatives lead by the International Human Genome Sequencing Consortium and Celera Genomics [63, 64]. However, the Sanger method has been partially supplanted by several “next-generation” sequencing technologies that offer dramatic increases in cost-effective sequence throughput, albeit at the expense of read lengths. The next-generation technologies commercially available today include the 454 GS20 pyro sequencing based instrument (Roche Applied Science), HiSeq (Illumina, Inc.), the SOLiD instrument from Applied Biosystems, and the Heliscope from Helicos, Inc. The reported read lengths of these technologies vary from 50-400 bp, although these figures are frequently changing.

Current “Next-generation” or “2^nd^ Generation” sequencing essentially relies on a modified “shotgun” approach, involving multiple steps: template fragmentation and amplification, sequencing, imaging and alignment of the sequences to a reference genome. A key aspect is that current technology is unable to sequence much more that 1000 base pairs in a single reaction; indeed much of the published data is derived from very short sequencing “reads” of around 35bp, something that can cause problems with downstream analysis, as these short reads may align to multiple locations in the reference genome requiring computationally expensive alignment algorithms. Artefacts can also be introduced at various steps, particularly during PCR amplification of the template fragments. If personalised genomics is to be realised we also need to ensure that protocols are standardised to minimise the run-to-run, and lab-to-lab variation that has been reported [65].

## FUTURE DIRECTIONS

### Genome Enrichment

In the short to medium term, research groups with a finite budget have to make the decision whether to perform whole genome-sequencing on a small number of subjects, or exome-sequencing on a larger number, weighing up the relative merits of each along the way. Use of exome-seq has recently been supported by a report that the entire CCDS (a database of consensus coding sequences in human and mouse) exome can be interrogated with just 2.8 Gbp of sequence data, approximately 3% of the required data for whole genome shotgun experiments [66] (although this still does not mitigate against the inability to identify variation in regulatory elements in non-coding regions).

### 3^rd^ Generation Technology

Second generation technology continues to evolve, with the main players continually improving both the hardware, and the chemistry that underpins sample preparation and the actually sequencing reaction itself. But already there are a number of technologies (the so-called 3^rd^ Generation sequencers) that may well deliver on the promise to provide the $1000 genome. Single-molecule, real-time sequencing negates the need for amplification, removing one of the key sources of bias mentioned above. Companies such as Helicos and Pacific Biosciences [67-69] are leading the way. In addition to the single-molecule nature of these technologies, read length is significantly increased, improving the ability of the analytical software to successfully map the reads back to the reference genome. Finally, single molecule sequencing can detect methylation at single-base resolution allowing the integration of genetic and epigenetic data [70].

## PERSPECTIVE: CLINICAL IMPACT

Phenotype based approaches largely govern the current practice of cardiovascular medicine. The influence of genetic variants is expected to correlate inversely with the proximity of the phenotype to the genes. Genetic determinants of the proximal (biochemical) phenotypes could help unravel the biological and functional sequence of DNA coding variants and might be translated to the clinical phenotype in the future. Whole genome sequencing is likely to become readily available at a reasonable cost and the genome/exome data could link genotype to the phenotype prospectively. The challenge would be to differentiate the associated alleles from the disease causing variants by means of genetic and biological studies.

Despite the very large number of functional alleles identified by GWAS and the rapidly evolving field of functional genomics, their application in clinical cardiovascular medicine is limited at present. However, these approaches have led to an improvement of our understanding of CVD and can be translated to clinical utility. Gene-based pre-symptomatic prediction of illness, finer diagnostic sub-classifications and improved risk assessment tools will permit earlier and more targeted intervention. Pharmacogenetics will guide our therapeutic decisions and monitor response to therapy. Personalised medicine will require the integration of clinical information, stable and dynamic genomics, and molecular phenotyping.

These have already found a place in cardiovascular screening, diagnosis and pharmacogenesis. As recently reviewed by Kim *et al.*, disease-causing mutations for a wide range of Mendelian cardiac disorders have been revealed through linkage studies, thereby permitting screening of family members to identify mutation carriers for early intervention [71]. A more controversial task is the use of common genetic variants identified in genome-wide association studies for risk prediction in the primary prevention of cardiovascular disease. Risk prediction using genomic information is still developing and the various predictors identified in GWAS are potential genomic predictors to cardiovascular risk. This is likely to improve in the future by inclusion of rare DNA variants identified by sequencing and incorporating functional genomic data into predictive models including epigenetic markers, transcriptomics and metabolomic biomarkers [72]. 

Non-invasive alternatives that reduce the need for invasive testing are a lucrative goal in clinical cardiology. Already in clinical use is the non-invasive diagnosis of cardiac allograft rejection with blood gene expression. The Invasive Monitoring Attenuation through Gene Expression: IMAGE trial compared the routine use of endomyocardial biopsies for monitoring rejection with a more selective use of endomyocardial biopsy guided by a gene-expression profiling test called AlloMap and noninvasive cardiac imaging. Both strategies resulted in equivalent clinical outcomes, but patients who were monitored with gene-expression profiling underwent far fewer biopsies per person-year of follow-up than did patients who were monitored with routine biopsy (0.5 versus 3.0; *P*_0.001) [73].

Pharmacogenomics is the study of how genetic variation affects the clinical response to drugs, with the implicit assumption that pharmacogenomic insights will enhance efficacy and reduce toxicity. It is likely to be the field that will benefit the most in the short term as it is easy to link genetics to the ability to metabolise a drug. Recognising that 25% of patients have a sub-therapeutic antiplatelet response to clopidogrel, researchers have identified several genetic variants affecting the metabolism of clopidogrel, a prodrug, to its active metabolite. Of these, the CYP2C19 variant allele has been best linked to impaired clopidogrel metabolism, reduced platelet inhibition, and a higher risk of adverse cardiovascular events after percutaneous coronary interventions [74]. Because of the cumulative data, the Food and Drug Administration has now altered the prescribing information for clopidogrel based on CYP2C19 genotype, a move that foreshadows the development of companion diagnostic testing and alternative inhibitors of ADP-mediated platelet activation that do not require metabolism by CYP2C19 [74]. In heart failure therapeutics, pharmacodynamic studies and post hoc analyses from clinical trials indicate that polymorphisms in the β1-adrenergic receptor affect the clinical actions of β-blockers [75, 76] whereas more limited data suggest common variants that affect therapeutic response in heart failure [77] and hyperlipidemia [78-80]. Warfarin is a widely used oral anticoagulant with several important indications, significant risks associated with either under-dosing or overdosing, and great patient-to-patient variability in dosing. Although many clinical and environmental factors are known to affect warfarin dosing, [81] several studies have identified common sequence variants in at least 2 genes (CYP2C9 and VKORC1) that strongly affect the pharmacokinetics and pharmacodynamics of warfarin [82-84]. Small clinical trials suggest that that genetically based warfarin-dosing algorithms may enhance the efficiency and safety of warfarin dosing [85, 86].

Hence, clinical applications of genomics are already enhancing the practice of cardiovascular medicine. Advances in functional genomics will be increasingly important to practicing cardiovascular specialists in the coming years.

### Personalised Medicine/Genomics

Although the driving force behind GWASs was originally the discovery of novel biological pathways through the use of scalable genome technology and its capacity for generating hypothesis-independent genetic associations, the results have generated much excitement about the potential clinical applications of these genetic markers for disease prediction, prevention and diagnosis. It is hoped that genomic discoveries would lead to personalised medicine, whereby healthcare interventions would be guided based on individual’s genomic make up. Direct-to-consumer genetic testing companies now offer mutation analysis and SNP microarray sequencing based on the findings of traditional genetic research and GWASs before the clinical validity or utility of population screening is understood. The challenge will be to reconcile people’s concerns about genomic privacy and security with the need to allow researchers and clinicians data access. Other implications including life/health insurance, psychological risks to the patients if they know they have a genetic variant (that means a significant increase in risk) and family screening have to be considered as well. Conversely, knowing early in life of the predisposition to certain conditions, could lead to lifestyle modification, risk prevention and early intervention.

Personalised medicine within cardiovascular medicine, has been emphasised in risk prediction models, especially coronary artery disease, where the goal is to identify individuals at risk so that early intervention and lifestyle modification can be initiated. Genotype based risk prediction is fixed from birth, allows early risk prediction, is less susceptible to biological variation over life and is easy to obtain with minimal measurement error. A series of recent studies attempted to demonstrate predictive utility of the 9p21 risk allele [87-96]. All showed significant association between the risk alleles and incident events, but only one showed an improvement in the C-statistic based on traditional risk factors [89]. 

As we discern more genomic markers–SNPs, copy number variants, and rare alleles– that influence the development and course of cardiovascular disease, our goal as evidence-based clinicians will be to apply this knowledge judiciously and responsibly for more personalised and cost-effective care.

Currently, there is an expanding gap between the availability of direct-to- consumer whole genome testing and physician knowledge regarding interpretation of test results. Advances in the genomic literacy of health care providers will be necessary for genomics to fulfil its potential to affect clinical practice.

## Figures and Tables

**Table 1.** GWAS Loci for CAD

Region	Reported Gene(s)	SNP	Risk Allele	P	Ref	OMIM Ref	GWAS Identifier
1p32.3	PCSK9	rs11206510	T	9.10×10^–8^	17	607786	HGVST11
1p13.3	SORT1	rs599839	A	2.89×10^–10^	18	*602458	HGVST11
1q41	MIA3	rs17465637	C	1.36 × 10^–8^	18	613455	HGVST148
2q33.1	WDR12	rs6725887	C	1.12 × 10^–9^	17		
3q22.3	MRAS	rs2306374	C	3.34 × 10^–8^	19	*608435	HGVST93
6p24.1	PHACTR1	rs12526453	C	1.15 × 10^–9^	17	*608723	HGVST11
6q25.3	LPA	rs3798220	C	3.0x10^–11^	20	152200	HGVST75
9p21.3	CDKN2A,	rs4977574	G	1.35 x10^–22^	18,21,22	*600160	HGVST11
	CDKN2B						
10q11.21	CXCL12	rs1746048	C	2.93 × 10^–10^	18	600835	HGVST11
12q24.12	SH2B3	rs3184504	T	6.35 × 10^–6^	23	605093	
19p13.2	LDLR	rs1122608	G	9.73 × 10^–10^	17	606945	HGVST75
21q22.11	MRPS6	rs9982601	T	4.22 × 10^–10^	17	*611973	
1p32.2	PPAP2B	rs17114036	A	3.81 × 10^–19^	15	*607125	
6p21.31	ANKS1A	rs17609940	G	1.36 × 10^–8^	15	*608994	
6q23.2	TCF21	rs12190287	C	1.07 × 10^–12^	15	*603306	HGVST11
7q32.2	ZC3HC1	rs11556924	C	9.18 × 10^–18^	15		
9q34.2	ABO	rs579459	C	4.08 × 10^–14^	15	110300	HGVST11
10q24.32	CYP17A1, CNNM2	rs12413409	G	1.03 × 10^–9^	15	*609300	HGVST11
	NT5C2					*607803, *600417	
11q23.3	ZNF259, APOA5-	rs964184	G	1.02 × 10^–17^	15	*603901	HGVST11
	A4-C3-A1				15	*606368	
13q34	COL4A1	rs4773144	G	3.84 × 10^–9^	15	*120130	HGVST75
	COL4A2					*120090	HGVST11
14q32.2	HHIPL1	rs2895811	C	1.14 × 10^–10^	15		
15q25.1	ADAMTS7	rs3825807	A	1.07 × 10^–12^	15	*605009	HGVST11
17p13.3	SMG6, SRR	rs216172	C	1.15 × 10^–9^	15	*610963, *606477	HGVST11
17p11.2	RASD1, SMCR3,	rs12936587	G	4.45 × 10^–10^	15	*605550	
	PEMT			1.81 × 10^–8^	15	*602391	
17q21.32	UBE2Z, GIP,	rs46522	T			*137240	HGVST11
	ATP5G1, SNF8					*603192, *610904	HGVST11
10q23.31	LIPA	rs1412444	T	6.29 × 10^–4^	16	*613497	HGVST11
		rs2246833	T	6.78 × 10^–4^	16		

**Table 2.** GWAS Loci for QRS Duration

Locus	Chromosome	Reported Gene(s)	SNP	Risk Allele	P	Ref	OMIM Ref	GWAS Identifier
1	3	SCN10A	rs6801957	T/C	1.10 ×10^–28^	37	*604427	HGVST489
	3	SCN10A-SCN5A	rs9851724	C/T	1.91 ×10^–20^	37	*600163	HGVST489
	3	SCN5A/EXOG	rs10865879	T/C	1.10 × 10^–28^	37	*604051	
	3	SCN5A	rs11710077	T/A	5.74 × 10^–22^	37	*600163	HGVST489
	3	SCN5A	rs11708996	C/G	1.26 × 10^–16^	37	*600163	HGVST489
	3	EXOG	rs2051211	G/A	1.57 × 10^–8^	37	*604051	
2	6	CDK1NA	rs9470361	A/G	3.00 × 10^–27^	37		
3	6	C6orf204-SLC35F1	rs11153730	C/T	1.26 × 10^–18^	37		
		PLN-BRD7P3				37	*172405	
4	1	NFIA	rs9436640	G/T	4.57 × 10^–18^	37	*600727	HGVST658
5	5	HAND1-SAP3OL	rs13165478	A/G	7.36 × 10^–14^	37	*602406	HGVST658
6	7	TBX20	rs1362212	A/G	1.12 × 10^–13^	37	*606061	
7	14	SIPA1L1	rs11848785	G/A	1.04 × 10^–10^	37		
8	12	TBX5	rs883079	C/T	1.33 × 10^–10^	37	*601620	HGVST489
9	12	TBX3	rs10850409	A/G	3.06 × 10^–10^	37	*601621	
10	10	VTI1A	rs7342028	T/G	4.95 × 10^–10^	37		
11	18	SETBP1	rs991014	T/C	6.20 × 10^–10^	37	*611060	
12	2	HEATR5B-STRN	rs17020136	C/T	1.90 × 10^–9^	37		
13	3	TKT-PRKCD-CACNA1D	rs4687718	A/G	6.25 × 10^–9^	37	*606781	
14	2	CRIM1	rs7562790	G/T	8.22 × 10^–9^	37	*606189	
15	1	C1orf185-RNF11	rs17391905	G/T	3.26 × 10^–10^	37	*612598	
		CDKN2C-FAF1				37	*603369	
16	17	PRKCA	rs9912468	G/C	1.06 × 10^–8^	37	*176960	HGVST658
17	7	IGFBP3	rs7784776	G/A	1.28 × 10^–9^	37	*146732	
18	1	CASQ2	rs4074536	C/T	2.36 × 10^–8^	37	*114251	HGVST658
19	13	KLF12	rs1886512	A/T	1.27 × 10^–8^	37	*607531	HGVST658
20	3	LRIG1-SLC25A26	rs2242285	A/G	1.09 × 10^–8^	37	*611037	
21	10	DKK1	rs1733724	A/G	3.05 × 10^–8^	37	*605189	
22	17	GOSR2	rs17608766	C/T	4.75 × 10^–10^	37	*604027	HGVST658

**Table 3.** GWAS Loci for Stroke

Disease	Region	Reported Gene(s)	SNP	Risk Allele	Ref	OMIM Ref	GWAS Identifier
All stroke	12p13.33	NINJ2	rs12425791	A	44	*607297	HGVST302
Intracranial aneurysm	2q33.1	BOLL, PLCL1	rs700651	G	44	*606165, *600597	HGVST98
Intracranial aneurysm	8q11.23	SOX17	rs10958409	A	44	*610928	HGVST14
			rs9298506	A	44		
Ischaemic Stroke	9p21.3	CDKN2A, CDKN2B	rs1333040	T	44	*600160	HGVST14
Ischaemic Stroke	4q25	PITX2	rs220073	T	44	*601542	HGVST14

**Table 4.** GWAS Loci for BP

Chromosome	Reported Gene	SNP	Risk Allele	P	Ref	OMIM	GWAS Identifier
1	MOV10	rs2932538	G/A	2.9 × 10^–7^	57	*610742	HGVST9
3	SLC4A7	rs13082711	T/C	3.6 × 10^–4^	57	*603353	HGVST9
3	MECOM	rs419076	T/C	3.1 × 10^–4^	57		
4	SLC39A8	rs13107325	T/C	4.9 × 10^–7^	57	*608732	
4	GUCY1A3–	rs13139571	C/A	2.5 × 10^–5^	57	*139396	HGVST9
	GUCY1B3					*139397	HGVST9
5	NPR3–	rs1173771	G/A	3.2 × 10^–10^	57	*108962	HGVST9
	C5orf23						
5	EBF1	rs11953630	T/C	1.7 × 10^–7^	57	*164343	HGVST9
6	HFE	rs1799945	G/C	1.8 × 10^–10^	57	*613609	HGVST9
6	BAT2–BAT5	rs805303	G/A	1.1 × 10^–10^	57	*142620	
10	CACNB2(59)	rs4373814	G/C	8.5 × 10^–8^	57	*600003	HGVST9
10	PLCE1	rs932764	G/A	3.2 × 10^–10^	57	*608414	HGVST9
11	ADM	rs7129220	G/A	9.4 × 10^–9^	57	*103275	HGVST9
11	FLJ32810–	rs633185	G/C	1.1 × 10^–3^	57		
	TMEM133						
15	FURIN–FES	rs2521501	T/A	5.4 × 10^–11^	57	*190030	HGVST9
17	GOSR2	rs17608766	T/C	7.0 × 10^–7^	57	*604027	HGVST9
20	JAG1	rs1327235	G/A	4.6 × 10^–4^	57	*601920	HGVST9
20	GNAS–EDN3	rs6015450	G/A	4.2 × 10^–14^	57	*139320	HGVST9
1	MTHFR–	rs17367504	G/A	2.3 × 10^–10^	57	*607093	HGVST9
	NPPB						
3	ULK4	rs3774372	T/C	0.18	57		
4	FGF5	rs1458038	T/C	1.9 × 10^–7^	57	*165190	HGVST9
10	CACNB2(39)	rs1813353	T/C	6.2 × 10^–10^	57	*600003	HGVST9
10	C10orf107	rs4590817	G/C	9.8 × 10^–9^	57		
10	CYP17A1–	rs11191548	T/C	1.4 × 10^–5^	57	*609300	HGVST9
	NT5C2						
11	PLEKHA7	rs381815	T/C	3.4 × 10^–6^	57	*612686	HGVST9
12	ATP2B1	rs17249754	G/A	1.1 × 10^–14^	57	*108731	HGVST9
12	SH2B3	rs3184504	T/C	2.6 × 10^–6^	57	*605093	
12	TBX5–TBX3	rs10850411	T/C	5.2 × 10^–6^	57	*601620	HGVST9
15	CYP1A1–	rs1378942	C/A	1.0 × 10^–8^	57	*108330	HGVST9
	ULK3						
17	ZNF652	rs12940887	T/C	1.2 × 10^–7^	57	*613907	HGVST9

**Table 5.** GWAS Loci for TAAD

Chromosome	Reported Gene	SNP	Risk Allele	P	Reference	OMIM
15q21.1	FBN1	rs10519177	G	2.6 × 10^–11^	58	*134797
15q21.1	FBN1	rs4774517	A	3.8 × 10^–11^	58	*134797
15q21.1	FBN1	rs755251	G	3.2 × 10^–11^	58	*134797
15q21.1	FBN1	rs1036477	G	6.5 × 10^–12^	58	*134797
15q21.1	FBN1	rs2118181	G	5.9 × 10^–12^	58	*134797
